# Silence in Conversations About Advancing Pediatric Cancer

**DOI:** 10.3389/fonc.2022.894586

**Published:** 2022-06-29

**Authors:** Sarah L. Rockwell, Cameka L. Woods, Monica E. Lemmon, Justin N. Baker, Jennifer W. Mack, Karen L. Andes, Erica C. Kaye

**Affiliations:** ^1^ Rollins School of Public Health, Emory University, Atlanta, GA, United States; ^2^ St. Jude Children’s Research Hospital, Memphis, TN, United States; ^3^ School of Medicine, Duke University, Durham, NC, United States; ^4^ Dana-Farber Cancer Institute, Boston, MA, United States; ^5^ Boston Children’s Hospital, Boston, MA, United States

**Keywords:** silence, communication, pediatric, cancer, medical education

## Abstract

**Background and Objectives:**

Skillful use of silence by clinicians can support patient-centered communication. However, what makes a period of silence feel meaningful is not well understood. This study aimed to characterize profound, skillful silences during difficult conversations between pediatric oncologists, children with advancing cancer, and their families.

**Methods:**

We audio-recorded serial disease reevaluation discussions between pediatric oncologists, patients with high-risk cancer, and their families across 24 months or until death, whichever occurred first. Using an inductive process, we performed content analysis across all dialogue recorded at timepoints of disease progression to examine types of silence.

**Results:**

17 patient-parent dyads with disease progression yielded 141 recorded conversations. Inductive coding yielded a layered typology of silence, including “intentional silence” (≥5 seconds), “profound silence” (≥5 seconds following receipt of difficult information, juxtaposed with statements of shared understanding, emotion, or enlightenment), and “stacked silence” (series of silences juxtaposed within dialogue). Intentional silence lasting ≥5 seconds occurred 238 times in 35/49 “bad news” recordings; nearly half (103/238) of these silences were identified as profound silence, in which silences appeared to create space for processing, allowed for questions to emerge, and synergized with empathic and affirmational statements. In most cases, profound silences involved the juxtaposition, or stacking, of multiple silences close together.

**Conclusions:**

Profound silences occur often during conversations about advancing pediatric cancer and share distinct characteristics. Opportunities exist to teach clinicians to use profound and stacked silences with intention during difficult conversations as a fundamental aspect of communication.

## Introduction

Children with cancer and their families face physical, psychosocial, and spiritual distress across the illness course. Honest, direct communication between patients, families, and clinicians is essential for provision of holistic, person-centered care during this stressful time (1–5). Use of silence is recognized as an integral aspect of empathic communication; silence serves as a mechanism for conveying support and respect, facilitating reflection, and bearing witness (6). In the context of suffering and difficult conversations, certain types of silence can create pivotal moments of shared understanding, connection, and presence (7). The use of empathic and clear communication punctuated by meaningful silence can build therapeutic alliance, reduce stress, and improve stakeholders’ perceptions of patient- and family-centered care (1).

Yet not all silence is equal. Silence defined as an absence of speech alone may entail awkward moments between clinicians and patients or be interpreted negatively by stakeholders (8). Within the field of communication science, researchers have examined differences between silences that engender connection, distance, or neutrality within patient-clinician encounters (7, 8). In general, connectional silences occur rarely, while silences that represent distance and neutrality are more common (7, 9). Silences that engender a sense of connection often feel profound and have been described within social sciences as “the stillness of listening to humanity”. (10)

Within medical research, the qualities and impact of profound silence as a communication tool remain understudied. In the field of pediatric cancer specifically, few studies have examined the characteristics of meaningful periods of silence within clinical encounters (7–9). The U-CHAT (Understanding Communication in Healthcare to Achieve Trust) trial was designed to better understand patterns in prognostic communication across advancing illness. In this paper, data from the UCHAT trial were analyzed to characterize the frequency and nature of silence in conversations about disease progression in advancing pediatric cancer.

## Materials and Methods

This study was conceptualized and developed by an interdisciplinary team of pediatric oncology and palliative care clinicians and researchers in collaboration with an institutional Bereaved Parent Steering Council; it was approved by the Institutional Review Board at St. Jude Children’s Research Hospital [U-CHAT (Pro00006473); approval date: 7/12/2016]. We present study methods and findings following the COREQ (COnsolidated Criteria for REporting Qualitative Research) checklist (**Supplemental Table 1**) (11).

Details about study recruitment, enrollment, and data collection processes were previously published (4, 12). Briefly, we enrolled a convenience sample of 33 children with high-risk cancer, their parents, and their primary pediatric oncologists at an academic pediatric cancer center.

Eligibility criteria and recruitment processes are summarized in **Table 1**. We followed patient-parent dyads prospectively and audio-recorded serial disease reevaluation discussions occurring in the clinic or hospital setting across each patient’s illness course until death or 24 months from disease progression on study, whichever occurred first. Demographic and disease-related information were extracted from the electronic medical record.

**Table 1:** Eligibility criteria, recruitment, and informed consent processes.

Protocol domain	Study information
Eligibility Criteria	Eligible oncologists: Primary oncologists providing medical care to solid tumor patients at the institution. Eligible patients: Aged 0-30 years, solid tumor diagnosis with survival of ≤50% estimated by their primary oncologist, projected to have ≥ 2 future time points of disease reevaluations. Eligible parents/guardians: Legal caregiver of eligible patient, aged ≥18 years, English language proficiency, planned to accompany patient to medical visits.
Recruitment & Informed Consent	Eligible primary oncologists were introduced to the study by the Principal Investigator (PI). The PI individually sent emails to eligible oncologists to determine interest in participating; once interest was expressed, the PI met one-on-one with oncologists to describe the study and complete the informed consent process. No oncologists declined participation. Eligible patient/parent dyads were identified by the research team through review of outpatient clinic schedules and institutional trial lists. The PI reviewed identified patients to determine those with overall survival estimated at 50% or less. A member of the research team then asked the patient’s primary oncologist: “In your clinical judgement, would you estimate [patient name]’s overall survival at 50% or less?” Permission to approach eligible dyads was requested from the primary oncologist. Patient-parent dyads were approached by a member of the research team during a clinic visit to determine interest in participation. If interested, the study was described in detail. Dyadic enrollment required agreement from both patient and parent. Patients aged ≥12 years provided assent, and patients aged ≥18 years and parents provided consent. Any other clinicians or individuals (e.g., family or friends of the patient) who planned to join a recorded conversation were approached by a member of the research team to learn about the study; following informed consent processes, verbal consent was obtained for the presence of study non-participants.

To better understand the landscape of silence as a facet of communication during difficult conversations, we analyzed all conversations at timepoints of disease progression (i.e., “bad news” conversations). A research team representing medical and nursing disciplines across pediatric oncology and palliative medicine (**Supplemental Table 2**) first reviewed the literature on silence as a communication approach within cancer care. Finding little consensus for fundamentals of connectional silence in pediatric cancer care, we used an inductive approach (13) to generate a typology for silences found within these conversations. Briefly, two researchers (S.R., E.K.) repetitively listened to audio-recordings, conducted memo-writing, and used raw data to inform development of codes, code definitions, and salient examples (14). Additional researchers (C.W., J.B.) reviewed recorded content and provided feedback in iterative cycles of codebook development. The codebook was pilot tested across several recordings representing various communication styles to identify areas of variance. We did not calculate interrater reliability, given theory suggesting that quantifying variances undervalues the interpretative mission of qualitative analysis (15). However, all analysts met regularly to review, discuss, and reconcile variances to achieve consensus, modifying the codebook when needed to improve dependability, confirmability, and credibility of independent codes (16).

The codebook was finalized following deep review of sufficient raw data to reach saturation, with no new concepts emerging from the recorded dialogue. Three levels of silence emerged from this process to comprise the codebook. First, intentional silence indicated an uninterrupted pause lasting at least 5 seconds; lapses in conversation in the context of transitions between topics or activities were excluded. This threshold was chosen following iterative memoing that revealed that pauses less than 5 seconds often were challenging to define conclusively as intentional. Second, profound silence emerged as a type of a silence in which the preceding or subsequent 60 seconds of dialogue included painful prognostic information, expressions of emotion, or shared sense of enlightenment. Third, stacked silence comprised a series of silence codes juxtaposed closely within dialogue, with fewer than 90 seconds between the end of one silence and the beginning of the following silence. Codes were not mutually exclusive, allowing for one or more codes to be applied to a given pause. The complete set of silence codes is presented in **Table 2**, alongside boundary examples of silences that met one criterion but not others to bolster understanding of the proposed taxonomy.

**Table 2:** Inductive Silence Codebook.

Code	Definition
Intentional Silence	Code for any uninterrupted pause that is 5 seconds or longer; a pause with intention, not including transitions. *Boundary examples for silences that meet 5 second threshold criterion, but not intentional criterion:* -Non-verbal pauses with ancillary distraction sounds (e.g., shuffling papers, rustling sounds as someone moves from one location to another, etc.) -Non-verbal pauses that come after statements indicating the need for a momentary pause (e.g., “Give me a sec to look up these results,” or “Let me just find the right spot on the image,” etc.) -e.g., Oncologist: “I’m amazed that she’s been doing so well and this stuff hasn’t gotten worse.* [>5 seconds of s*ilence, punctuated by rustling food wrappers] Ok. Other questions?*” Patient: “No.”*
Profound Silence	A coded silence in which, during the preceding or following 60 seconds, the following occurs: The provider shares “bad news” information (or references having just given this information) to patient/family related to scan results OR treatment options OR progression of disease OR goals of care OR prognosis **AND** at least one element below (before or after silence code): Statements about shared understanding/acknowledgement between provider and family **OR** Statements or expressions of enlightenment/catharsis. Can include provider responding to a question or making a statement that includes an element of truth-telling **OR** Expression of emotion by patient/family which preceded or followed statement by provider giving indication of shared emotions/recognition of expressed emotions. Can include provider invitation to continue expression of emotion. *Boundary example for silence that meets the intentional criterion, but not the criterion for profound silence:* *Oncologist: So my opinion is even if she hasn’t shown marked improvement* *in the bony lesions, if her symptoms are actually really good … [>5 seconds* *of silence] … we may also be getting some control there as well.*
Stacked Silence	A series of **Silence** codes that are linked together as one segment of conversation, with no more than 90 seconds occurring between the end of one silence and the beginning of the following silence.

All processes were conducted within MAXQDA, a mixed methods data analysis software system (Verbi GMBH, Berlin, Germany) (17). Following codebook finalization, independent double coding of each recorded encounter was performed by two analysts (S.R., C.W.), with weekly meetings to review coding variances and third-party (E.K., J.B.) adjudication to reach consensus. Consistency in code segmentation was reviewed to ensure a standardized approach (S.R., C.W., E.K.). Content analysis of dialogue surrounding coded silence was conducted, identifying themes co-occurring with profound silence (S.R., E.K.) Quotations surrounding profound silence and stacked silence were examined to identify patterns in language, content, and timing of silence to generate themes (S.R., E.K.).

## Results

For the 17 patient-parent dyads who experienced advancing disease during the study period, 141 disease reevaluation conversations were audio-recorded, comprising approximately 2400 minutes of recorded dialogue. Of these, 49 recorded discussions occurred at a timepoint of disease progression and were subsequently analyzed. Participating patients were mostly female (64.7%) and white (88.2%); further participant demographic variables are presented in **Table 3**. A median of 7 medical discussions per patient were recorded (range 1-19). Most patients (14/17) died during the study period; 3 remained alive at 24 months. Data on patient-parent dyads who declined enrollment have been previously published; briefly 17% of approached dyads (n=7 dyads) did not enroll due to hesitation or refusal by either the patient (n=4) or parent (n=4). Although small numbers, refusal rates did not appear disproportionate by race or ethnicity (4, 12).

**Table 3:** Participating patient, parent, and oncologist characteristics.

Variable	n (%)
**Patient (n=17)**	
Gender	
Female	11 (64.7)
Male	6 (35.3)
Race	
White	15 (88.2)
Black	1 (5.9)
Mixed	1 (5.9)
Ethnicity	
Hispanic	0 (0)
Non-Hispanic	17 (100)
Age at Diagnosis	
0-2 years	2 (11.8)
3-11 years	6 (35.3)
12-18 years	7 (41.2)
19+ years	2 (11.8)
**Parent (n=17)**	
Gender/Role	
Female/mother	14 (82.4)
Male/father	3 (17.6)
**Pediatric Oncologist (n=6)**	
Gender	
Female	3 (50)
Male	3 (50)
Race	
White	6 (100)
Black	0 (0)
Ethnicity	
Hispanic	0 (0)
Non-Hispanic	6 (100)
Years in Clinical Practice	
1-4 years	2 (33)
5-9 year	2 (33)
10-19 years	0
20+ years	2 (33)

In 35 out of 49 conversations (71%), periods of intentional silence were identified 238 times. Nearly half of these silences (103/238) were dual coded as profound silence. Profound silence length ranged from 5-102 seconds. Below, we describe features of profound silence and themes identified within dialogue preceding (i.e., prompting) and following (i.e., emerging from) moments of profound silence.

### Creating Space for Processing

Profound pauses seemed to create space for patients and families to absorb painful information. At times, after sharing difficult news (e.g., disease progression or relapse, poor prognosis, incurable illness), the oncologist would pause purposefully, as if to give the patient and family time to hear the message:

“So right now, we are really just going to try to find a drug that could potentially work, the likelihood of that happening is relatively low, but we can try a variety of experimental agents to see if they can work against this tumor. [Patient: Could I die]? You could potentially die from this, yes. **Silence-21 seconds.**

### Offering Opportunity for Questions

Silence also appeared to create opportunities for questions from patients or families, which may not have been voiced without a prolonged nonverbal space. For example, in the following conversation, the oncologist paused after affirming a family’s goals of care. Out of the resulting silence, the parent was able to formulate a question. When the oncologist continued to remain silent, the parent was able to verbalize and complete the emotionally challenging question:

“And you’ve been working on this so hard. And you deserve to do those things. You deserve to get to go to prom and go to Disney world and all of those kinds of things. And part of our goal should be to help you do that. Absolutely. [Parent: If we don’t [do treatment] … ] **Silence-6 seconds.** [Parent: Is she goin’ to hurt]? **Silence-8 seconds**.”

### Welcoming Empathic Statements

Many profound silences included an empathic statement by the oncologist, often emerging from the silence. Just as silence invited emotional expression to emerge or persist, short empathic utterances also created space for audible emotion to be held. In one conversation, the oncologist sat in silence while a parent cried, then offered brief condolences before reentering silence:

“But I wouldn’t probably do that [go on a trip] until the radiation is done. [Parent: Yeah. After the radiation is finished]? Assuming she feels good enough, that would be the time to try that. **Silence-8 seconds**. [parent audibly crying] I’m sorry. **Silence-37 seconds**. [parent audibly crying].”

At other times, expressions of empathy preceded silence, first acknowledging the grief and then bearing witness: “*I’m sorry sweetheart, I wish I had better news for you today.* **Silence-10 seconds** *[Patient Audibly Crying].”* In a different conversation, another oncologist expressed empathy before entering silence, creating space for shared grief:

“I know that she is very resilient and that she is very positive, and that she is probably in denial. Which is perfectly understandable. But I know she doesn’t feel good. [Parent: I know she doesn’t feel good. [audibly crying.]] She just doesn’t look the way she normally does. [Grandparent: I think she has lost a lot of her fighting spirit too.] I’m sorry. **Silence-10 seconds**. [parent audibly crying].”

### Emphasizing Affirmation

Within spaces of profound silence, oncologists offered statements to affirm or validate different choices about treatment and goals of care made by the patient or family. In many cases, affirming statements occurred at the onset of a profound silence, where the silence that followed the statement further reinforced the authenticity of the affirmation. For example, an oncologist validated a family’s prior interest in shifting focus to quality of life:

“There is even the choice to focus less on the [cancer] itself and focus more on you and how you’re feeling every day when you get up. So that you know you’re maximizing, you know you’re maximizing the way that you feel and that you can get the most out of every day … And you know none of those are wrong choices, it’s just a matter of what, at this point, what you feel is the most important goals for you … **Silence-26.7 seconds.** [patient audibly crying].”

Oncologists also used silence to affirm their role as a partner and supporter across the illness course:

“We really want you to come up with that, come up with lists so we can together all make the best decisions for you guys. With you guys, not for you, it’s with you. Okay? **Silence-7 seconds**. [parent audibly sniffling]. I’m sorry. **Silence-5 seconds**.”

### Stacking Silence

Multiple silences often occurred in close proximity with one another, and approximately three-quarters of profound silences within “bad news” conversations involved stacked silences. We found a wide range of stacking, from two distinct silences occurring within 90 seconds of dialogue to up to 12 distinct silences occurring within 8 minutes of dialogue. In most stacked silence moments, approximately one silence occurred per minute of conversation to generate a series, evoking a rhythmic pattern to the conversation. In many cases, each subsequent silence helped to advance the conversation into further exploration of difficult topics:

“Would you like for us to talk to [patient’s name]? Do you want to talk to her? Do you want her to talk? What would you like us to do? [Parent: Well, you decide amongst yourselves]. **Silence-5 seconds**. Can you tell me what you think would be the most appropriate thing to do?…If we say we are going to try something else then we are already committing to something we don’t know if we are going to do or not. [Parent: Okay]. Would you like us to tell her that? Would you like to tell her that? I’ll do whatever you want us to do. [Parent: Y’all can tell her]. **Silence-7 seconds**. I’m very sorry. **Silence-22 seconds**.”

In some cases, multiple silences were stacked within a few seconds of each other, with minimal conversation between silences. This phenomenon most commonly occurred when the oncologist spoke in brief phrases and allowed silence to dominate. In these scenarios, empathic statements typically punctuated a longer segment of silence, as previously described. These empathic interjections from the oncologist between silences often involved reassurance or bearing witness, with subsequent silence encouraging emotional expressions from the patient or family.

“What can I do for you? [Parent: You’ve done everything. I mean … [parent audibly crying]]. **Silence-25 seconds**. [parent audibly crying] You’ve done everything too. **Silence-54 seconds**. [parent audibly crying]. I’m so sorry. **Silence-9 seconds**. [parent audibly crying].”

We also identified a pattern where oncologists juxtaposed open-ended questions with stacked silences, offering an invitation or opportunity for the patient or family to emerge from silence and continue a conversation at their own pace:

“I don’t think she can be cured. There may be a very, very slim possibility of controlling it with something. But I do not believe there is a cure for her disease unfortunately. **Silence-60 seconds**. [parent audibly sighing] What is going through your mind now? What questions do you have for us? **Silence-20 seconds**. [parent audibly sniffling] [Parent: I guess I just have to talk to her dad … ]. **Silence-8 seconds**. [parent audibly crying] [Parent: Every bit of the mom in me would lean toward the experimental … you know]. Of course, that’s understandable.”

## Discussion

High-quality communication between clinicians and patients and their families is essential to person-centered care, improving psychosocial outcomes while facilitating therapeutic alliance in pediatric oncology (5, 18–20). Although silence is considered a strategic aspect of communication, the landscape of silence during difficult pediatric cancer conversations remains understudied. In this paper, we identified periods of intentional silence occurring in more than two-thirds of difficult conversations about disease progression. Nearly half of intentional silences were recognized as meaningful, or profound, in their ability to create space for processing, allow questions to emerge, and acknowledge and affirm emotional expression. Interestingly, most profound silences also involved the stacking of multiple silences close together.

Patients and families place high value on their clinician’s recognition and affirmation of their emotions during difficult conversations (21–24). Prior data demonstrate that clinicians inconsistently respond to emotional cues and concerns expressed by patients and families (22, 25) and frequently verbally dominate conversations, affording less time for patients or families to speak or ask questions (26, 27). When clinicians speak less, however, more opportunities for emotional disclosure by patients and families manifest (22). Building upon these findings, this study suggests that skillful use of silence, particularly in synergy with empathic statements and stacking of silence, has the potential to foster meaning-making and connection between clinician, patient, and family.

Being purposeful about creating space for silence, however, does not necessarily encompass skillful use of silence. Intuitively, unintentional silences (e.g., shuffling papers, transitioning locations, etc.) do not offer opportunities for connection or meaning-making; one might think that intentional silences intrinsically create these opportunities. Yet inductive coding revealed distinct concepts showing intentional silence as a pattern distinct from other types of silence, suggesting that being purposeful (intentional) about silence does not necessarily make the silence meaningful.

In this study, profound and stacked silences appeared to facilitate a psychological space in which everyone in the room had an opportunity to sit together and process information. These data emphasize the importance of silence as a tool for encouraging processing of emotions as well as processing of cognitive information, both of which influence decision making processes (28). Intense emotional reactions, such as those elicited when hearing information about disease progression, can derail an individual’s capacity for processing and rational decision making (29). People experiencing intense emotions (sometimes described as “hot” states) make different choices compared to people in “cold” states (30), and the difference between a hot vs. cold state can impact person-centered decision making around treatment options and end of life care choices (28). Skillful use of silence by clinicians may give patients and families space for processing, allowing for further conversation and decision making to occur in a less “hot” state.

We advocate for silence to be taught as an integral aspect of communication training for clinicians. When faced with emotional expression by patients or families, clinicians most often respond with provision of information, which in turn decreases space for further emotional disclosure (22). Existing communication training programs emphasize provision of empathic statements and emotional support, but the specific importance of silence as a communication strategy is less often described in the literature (31, 32). When mentioned, silence generally is presented as a tool for creating space immediately after the provision of bad news (33), and additional opportunities exist to develop and explore experiential learning techniques to teach clinicians to integrate and stack silence with purpose across medical dialogue as a fundamental communication strategy.

Importantly, not all profound silences may be interpreted positively by patients or families; at times, silence during profound moments may be a default reaction when clinicians do not know what to say. Further research is needed to examine the impact of profound silence and stacked silence on therapeutic alliance and shared decision-making between clinicians, patients with serious illness, and their families.

This study has several limitations, including single site design, convenience sampling of participants, and limited racial and ethnic representation. All eligible oncologists participated, comprising a range of styles and years of clinical practice, however they are not necessarily representative of all oncologists’ practice styles and strategies. A few discussions were not recorded due to logistical issues or at the request of the participating patient or parent. Although missing data could influence synthesis and interpretation of silences, given saturation of themes across thousands of recorded minutes, several missing timepoints are less likely to impact synthesis of findings. Codes were inductively derived based on clinical experience and lacked input from patient and family perspectives, which is needed for further validation in future studies.

In summary, intentional integration of silence in conjunction with empathic statements may enhance processing of information and emotional expression, fostering a sense of connection and meaning-making during difficult conversations between oncologists and their patients/family. Stacking silence also may afford opportunities for engendering profound, connectional moments during challenging clinical encounters.

## Data Availability Statement

The raw data supporting the conclusions of this article will be made available by the authors, without undue reservation.

## Ethics Statement

The studies involving human participants were reviewed and approved by St. Jude IRB. Written informed consent to participate in this study was provided by the participants’ legal guardian/next of kin.

## Author Contributions

SR conceptualized this study, carried out the analyses, drafted the initial manuscript, and revised the manuscript. CW collected data, carried out the analyses, and critically reviewed the manuscript for important intellectual content. ML assisted with analysis and reviewed and revised the manuscript. JB conceptualized and designed the study, supervised data collection, and critically reviewed the manuscript for important intellectual content. JM conceptualized and designed the study, assisted with analysis, and reviewed and revised the manuscript. KA conceptualized and designed the study, supervised analysis, and critically reviewed the manuscript for important intellectual content. EK conceptualized and designed the study, coordinated and supervised data collection, carried out the analysis, drafted the initial manuscript, and revised the manuscript. All authors approved the final manuscript as submitted and agree to be accountable for all aspects of the work.

## Funding

This work is supported by EK’s Career Development Award from the National Palliative Care Research Center and by ALSAC. Additionally, ML receives salary support from the National Institute of Neurological Disorders and Stroke (K23NS116453).

## Conflict of Interest

The authors declare that the research was conducted in the absence of any commercial or financial relationships that could be construed as a potential conflict of interest.

## Publisher’s Note

All claims expressed in this article are solely those of the authors and do not necessarily represent those of their affiliated organizations, or those of the publisher, the editors and the reviewers. Any product that may be evaluated in this article, or claim that may be made by its manufacturer, is not guaranteed or endorsed by the publisher.

## Abbreviations

U-CHAT trial, Understanding Communication in Healthcare to Achieve Trust trial.
