# Supplementary material for: Silence in Conversations About Advancing Pediatric Cancer
Source: Front Oncol. 2022 Jun 29;12:894586. doi: 10.3389/fonc.2022.894586 (PMC9277146; doi:10.3389/fonc.2022.894586)
Supplement: Supplementary file 1 [file Table_1.docx]

Supplemental Table 1. CORE-Q (COnsolidated criteria for REporting Qualitative research) Checklist

A checklist of items that should be included in reports of qualitative research. You must report the page number in your manuscript where you consider each of the items listed in this checklist. If you have not included this information, either revise your manuscript accordingly before submitting or note N/A.

| Item No. | Topic | Guide Questions/Description | Reported on Page No. |
| --- | --- | --- | --- |
| **Domain 1: Research team and reflexivity** | | | |
| *Personal characteristics* | | | |
| 1 | Interviewer/facilitator | Which author/s conducted the interview or focus group? *No data from interviews or focus groups are presented in this manuscript.* | n/a |
| 2 | Credentials | What were the researcher’s credentials? *Although this manuscript does not present interviewer-facilitated data, we describe the credentials of the researchers who participated in this study.* | Supplemental Table 2 |
| 3 | Occupation | What was their occupation at the time of the study? *Similar as above, we describe the occupations of the researchers on the study team.* | Supplemental Table 2 |
| 4 | Gender | Was the researcher male or female? *We describe the genders of the researchers on the study team and provide initials for all researchers who participated in each stage of the study to enable readers to connect individuals with names within the authorship.* | Supplemental Table 2 |
| 5 | Experience and training | What experience or training did the researcher have? *We describe the experience and training of the researchers on the study team.* | Supplemental Table 2 |
| *Relationship with participants* | | | |
| 6 | Relationship established | Was a relationship established prior to study commencement? *No data from interviews or focus groups are presented in this manuscript, so there is no interviewer-interviewee relationship to query.* | n/a |
| 7 | Participant knowledge of the interviewer | What did the participants know about the researcher? (e.g., personal goals, reasons for doing the research) *See above. Researchers did not engage with participants with respect to audio-recording of disease reevaluation conversations or completion of surveys.* | n/a |
| 8 | Interviewer characteristics | What characteristics were reported about the interviewer/facilitator? e.g., bias, assumptions, reasons and interest in the research topic? *See above.* | n/a |
| **Domain 2: Study design** | | | |
| *Theoretical framework* | | | |
| 9 | Methodological orientation and theory | What methodological orientation was stated to underpin the study? e.g., grounded theory, discourse analysis, ethnography, phenomenology, content analysis. *Content analysis was used in this study.* | Page 9; Table 2 |
| *Participant selection* | | | |
| 10 | Sampling | How were participants selected? e.g., purposive, convenience, consecutive, snowball. *We used a convenience sample of oncologists who practice in the Solid Tumor clinic at a pediatric cancer center and their patients/families.* | Page 7; Table 1 |
| 11 | Method of approach | How were participants approached? e.g., face-to-face, telephone, mail, email. *We approached participants to gauge interest either by phone or face-to-face, and we completed formal consents in face-to-face discussions.* | Table 1 |
| 12 | Sample size | How many participants were in the study? *We enrolled 6 primary oncologists and 4-6 patient-parent dyads per oncologist; this study presents data for the 17 patient-parent dyads that experienced advancing disease while on study.* | Page 7; Table 3 |
| 13 | Non-participation | How many people refused to participate or dropped out? Reasons? *We present data related to participant refusal and associated rationale; no participants dropped out of the study, although 1 patient sought care at another institution prior to death so presumably further disease reevaluation discussions occurred and were not recorded outside of the study site.* | Page 9-10 |
| *Setting* | | | |
| 14 | Setting of data collection | Where was the data collected? e.g., home, clinic, workplace. *Medical conversations were recorded in the clinic or hospital setting.* | Page 7 |
| 15 | Presence of non-participants | Was anyone else present besides the participants and researchers? *Other clinicians and family or friends of the patient were present at times during recorded discussions.* | Table 1 |
| 16 | Description of sample | What are the important characteristics of the sample? e.g., demographic data, date. *Demographic data for participants are presented in Table 3; dates of medical discussions are not presented as these might be identifiers and are not relevant for interpretation of findings.* | Table 3 |
| *Data collection* | | | |
| 17 | Interview guide | Were questions, prompts, guides provided by the authors? Was it pilot tested? *No data from interviews or focus groups are presented in this manuscript.* | n/a |
| 18 | Repeat interviews | Were repeat interviews carried out? If yes, how many? *See above.* | n/a |
| 19 | Audio/visual recording | Did the research use audio or visual recording to collect the data? *Disease reevaluation discussions were audio-recorded in real time.* | Page 7 |
| 20 | Field notes | Were field notes made during and/or after the interview or focus group? *n/a.* | n/a |
| 21 | Duration | What was the duration of the interview or focus group? *n/a* | n/a |
| 22 | Data saturation | Was data saturation discussed? *We recorded all disease reevaluation discussions for participating dyads until death or 24 months since disease progression on study. In this context, saturation was not relevant in terms of stopping data collection. However, we describe how saturation was achieved with respect to coding processes.* | Page 7-8 |
| 23 | Transcripts returned | Were transcripts returned to participants for comment and/or correction? *We did not consent patients/parents to return transcripts or data synthesis, as this was not felt to be appropriate in the context of recorded medical dialogue (as opposed to interviews), particularly given that most children had died and parents were bereaved at study end.* | n/a |
| *Data analysis* | | | |
| 24 | Number of data coders | How many data coders coded the data? *We describe the number and role and identification of all data coders who participated in this study.* | Page 9 |
| 25 | Description of the coding tree | Did authors provide a description of the coding tree or codebook? *We present the codebook in Table 2.* | Table 2 |
| 26 | Derivation of themes | Were themes identified in advance or derived from the data? *Themes were inductively derived from raw data.* | Page 8 |
| 27 | Software | What software, if applicable, was used to manage data? *We used MAXQDA software.* | Page 9 |
| 28 | Participant checking | Did participants provide feedback on the findings? *We did not consent patients/parents to provide feedback on findings, as this was not felt to be appropriate given that most children had died and parents were bereaved at study end.* | n/a |
| *Reporting* | | | |
| 29 | Quotations presented | Were participant quotations presented to illustrate the themes/findings? Was each quotation identified? e.g., participant number. *Representative quotes are embedded within the text. Quotes are identified as oncologist or patient/parent.* | Pages 10-14 |
| 30 | Data and findings consistent | Was there consistency between the data presented and the findings? *We demonstrate consistency between data presented in the Results section and interpretation of findings delineated in the Discussion section.* | Pages 9-17 |
| 31 | Clarity of major themes | Were major themes clearly presented in the findings? *We presented all major themes in detail.* | Pages 9-14 |
| 32 | Clarity of minor themes | Is there a description of diverse cases or discussion of minor themes? *We provide a variety of rich quotes embedded within the text to offer readers diversity of cases.* | Pages 9-14 |

Developed from: Tong A, Sainsbury P, Craig J. Consolidated criteria for reporting qualitative research (COREQ): a 32-item checklist for interviews and focus groups. *International Journal for Quality in Health Care*. 2007. Volume 19, Number 6: pp. 349 – 357.

Supplemental Table 2. Research Team Attributes and Qualifications

| **Author** | Attributes and Qualifications |
| --- | --- |
| E.K. | Female physician-scientist with a Medical Degree, a Master’s in Public Health, graduate-level training in qualitative research methodology with a focus on communication science, and clinical training and practice in pediatric hematology-oncology and hospice and palliative medicine. |
| S.R. | Female nurse-scientist with a Master’s in Public Health, graduate-level training in qualitative research methodology, and clinical training and practice as a pediatric oncology nurse and a pediatric advanced practice provider. |
| C.W. | Female research associate with formal MAXQDA training and expertise in qualitative research methodology. |
| K.A. | Female scientist with a Ph.D. in qualitative research methodology and extensive experience with teaching and conducting qualitative research. |
| M.L. | Female physician-scientist with a Medical Degree, graduate-level training in qualitative research methodology with a focus on communication science, and clinical training and practice in pediatric neurology and neonatal neurology. |
| J.B. | Male physician-scientist with a Medical Degree, extensive clinical and research expertise related to difficult communication in oncology, and clinical training and practice in pediatric hematology-oncology and hospice and palliative medicine. |
| J.M. | Female physician-scientist with a Medical Degree, a Master’s in Public Health, extensive research expertise in communication science, clinical training in pediatric hematology-oncology and hospice and palliative medicine, and practice in pediatric hematology-oncology. |
